# Low carbon and environmentally friendly livestock. The Costa Rican approach

**DOI:** 10.1093/af/vfab036

**Published:** 2021-09-06

**Authors:** Johnny Montenegro, Sergio Abarca

**Affiliations:** Livestock Department, Climate Change Unit, Instituto de Innovación y Transferencia en Tecnología Agropecuaria, Costa Rica

**Keywords:** commercial farms, grazing, humid tropic, mitigation

**Implications**

- Public-private cooperation and coordination is a key point to achieve the transformation of livestock activity in Costa Rica.
- Grazing livestock farms have great potential to remove atmospheric carbon in different ways. The intensification of commercial livestock production systems is a way to reduce the emission intensity.
- A training program with multidisciplinary approach and concrete and affective actions have significantly contributed to achieve that producers could implement improvements in its farms.
- Livestock production systems adapted to the ecological conditions in which they are located is a Good way to mitigate climate change.

## Introduction

Livestock are an important source of greenhouse gases worldwide, and, therefore, campaigns are being developed to reduce their environmental impact. However, in tropical regions, pasture areas can retain significant amounts of carbon in biomass and in the soil.

Costa Rica, located in the tropics, decided to take advantage of this ecological condition, focusing on livestock development, not only to improve production efficiency but also to carry out actions that mitigate climate change.

## Support Policies

The implementation of a livestock Nationally Appropriate Mitigation Action (NAMA) in 2014 (MAG, 2015), the conceptualization and implementation of the National Decarbonization Plan 2018–2050, and the development of the National Strategy for Low Carbon Livestock in 2015 (MAG-MINAE, 2015) are policies that directly support livestock productivity while mitigating climate change.

## The Context

There is potential in grazing livestock farms to remove atmospheric carbon in three ways: keeping at least 21% of their area in rainforests, increasing soil carbon fixation by using improved tropical grass species, and growing trees within pasture areas. Approximately, 40% of the country’s territory belong to livestock farms (INEC, 2020) and 97% of them have grazing management.

**Figure 1.**
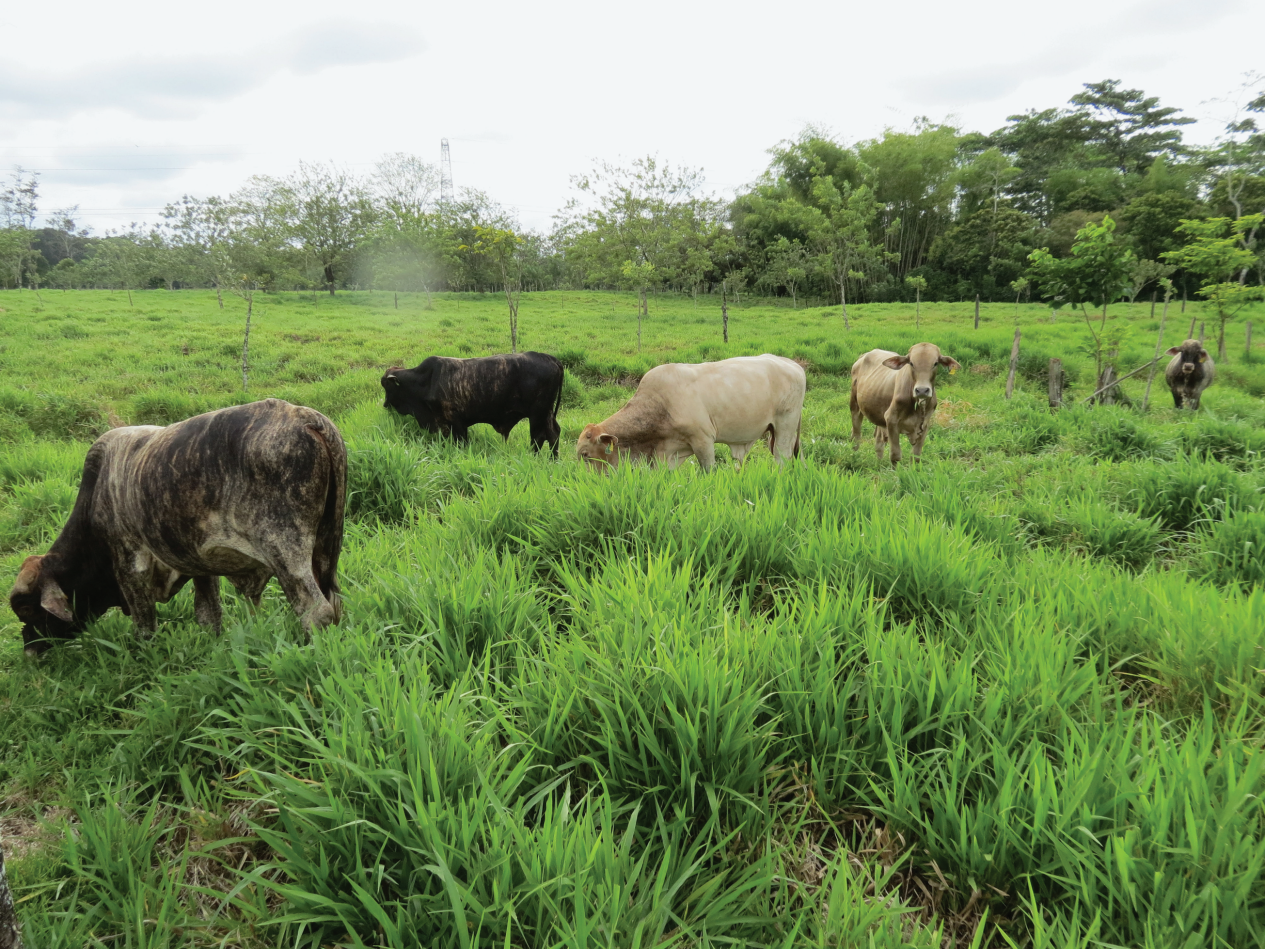
Cattle grazing in Costa Rica.

Also, there have been substantial efforts to reduce methane and nitrous oxide emissions in commercial farms mainly through a strong education program, which includes proper tropical pastures management, silvopastoral systems, improved grasses (Figure 1), and supplementation under grazing.

Currently, the Ministry of Agriculture and Livestock keeps records on 1,000 commercial farms that produce meat and milk. The trees within the pastures on the farms are geo-referenced and measured each year to document carbon retention. This component is particularly important since there are an estimated 23 million trees (MAG, 2021) in 1.3 million hectares of pasture (INEC, 2020).

## Soil Organic Carbon

More data are needed regarding soil carbon. Between 2017 and 2020, 21 farms distributed across the country were monitored, and data from 11 cow–calf and dual-purpose farms show significant amounts of stored ^13^C from of tropical grasses in the first 40 cm of depth (Abarca et al., 2018a). Experiments with hybrid grasses (*Urochloa* genus) in the very humid tropics show significant increases in organic carbon in well-managed pastures (Arguedas et al., 2020). However, it is necessary to detect any variation over time in the soil organic carbon in these commercial farms. Recent research has shown that soil carbon fixation is dependent on climate and soil (Montenegro and Barrantes, 2021). Efforts are currently being made to increase research in this line.

## Enteric Methane

Enteric methane is the main source of emission in livestock in Costa Rica. In monitored commercial farms this emission represents up to 99% of the total emitted Carbon dioxide equivalent (CO_2_eq) (MAG, 2021). For this reason, the livestock NAMA has prioritized production techniques and practices that reduce methane emissions; these include genetic improvement, diet quality, and changes in herd structure.

Emission estimates made with Tier II of IPCC (2006) methodology for Greenhouse gas (GHG) inventories have shown that, with the suggested pasture management (flexible regrowth period according to climatic season to achieve the best available quantity: quality ratio and short periods of permanence in the paddock), in addition to improving technical indexes that reduce unproductive times in the life of cattle (e.g., calving intervals and growth rate), it is possible to reduce enteric methane emissions by between 5% and 15%. This is especially important since these results are produced with management, without using additives or additional feed, which is why producers implement it without any inconvenience, while showing the potential that these simple management measures have for mitigation.

Because of this, investigations have been carried out to advance in the determination of the real methane emission in both meat and milk production systems (Montenegro et al., 2016, 2018, 2019; Abarca et al., 2018b; Soto and Abarca, 2018).

## Nitrous Oxide Emissions

The emissions from the primary livestock sector in the monitored farms represent less than 1% of the total, and, in the case of those coming from excreta (solid and liquid), it has been shown that are extremely low (Montenegro and Barrantes, 2019).

Data from the monitored farms in 2020 indicate that on average the quantity of carbon removals (captures + reductions) represented 63% of the emissions of the livestock system, expressed as CO_2_e (MAG, 2021).

## Training

A key aspect in this livestock strategy implemented in Costa Rica has been training both producers and technicians. The training strategy takes a multidisciplinary approach (animal nutrition, pasture and grazing management, soil conservation, animal genetics, production, and productivity) with concrete and effective actions. Although it has not been possible to follow up with trainees, reduced enteric methane emissions suggest that the hundreds of producers reached have been able to implement improvements in grazing systems.

## Target

The reduction of GHG emissions per unit of product obtained, meat or milk, is part of the scheme implemented in livestock operations in Costa Rica. The Research and Technology Transfer Program for Low Carbon Livestock in 2018 recommended reducing enteric methane emission targets for the participating farms of the NAMA that specialized milk production systems to below 20 g CH_4_ kg^−1^ milk. For beef cattle developed in Costa Rica and grazed exclusively tropical forages, a minimum daily live weight gain of 550 g, with no more than 170 g CH_4_ animal^-1^ day^-1^, so as not to exceed a methane conversion factor (Y_m_) of 5.0% is considered feasible to achieve.

## Conclusions

In Costa Rica, we consider bovine production based on forages and in silvopastoral systems, without deforestation, with adequate grazing management and diets based on forages. The use of animals adapted to the ecological conditions that prevail in the different regions where livestock production systems are located is a good option to mitigate climate change through low-emission livestock farming and good environmental practices while running productive operations at the same time.

## About the Authors

**Johnny Montenegro** is an Agronomist from Universidad de Costa Rica (UCR) and completed graduate degrees at Tropical Agricultural Research and Higher Education Center (CATIE), Master of Science (2001), and PhD (2011) at the University of Alberta, Canada. He has been a researcher in the areas of agroforestry and silvopastoral systems and, in the last 10 years, directly involved in climate change and agriculture research. His current research at the National Institute for Agricultural Transfer (INTA) in Costa Rica involves studies on the following: soil carbon, N_2_O, and enteric methane emissions. He participated in invited talks and different activities related with professional organizations, producers, and academics organized by national and international universities and organizations.

**Sergio Abarca-Monge** has been a researcher in the interaction environment/animal production for the humid tropics of Central America in the last 25 years. He has been the Director of the Zoosanitary and Phytosanitary Services of Costa Rica. He was coordinator of the Program for Research and Technology Transfer for Low-Carbon Livestock. He is currently the coordinator of Climate Change and Agriculture at the National Research Institute for Agricultural Transfer (INTA) of the Ministry of Agriculture and Livestock of Costa Rica. His studies are generally on the integration of silvopastpril systems as an alternative for the reduction of net carbon emissions and protection of biodiversity and water resources in regions with high rainfall.
